# Comparison of the Fetal Fraction of Cell-Free DNA in In-Vitro Fertilization (IVF) Versus Natural Conception Evaluation of the Fetal Fraction With IVF Parameters

**DOI:** 10.7759/cureus.24516

**Published:** 2022-04-26

**Authors:** Kostas Kallianidis, Evangelia Dimitroulia, Depy Mavrogianni, Emmanuaela Liokari, Ritsa Bletsa, Elli Anagnostou, Nikos Sofikitis, Dimitrios Loutradis

**Affiliations:** 1 Fertility Institute, National and Kapodistrian University of Athens School of Medicine, Athens, GRC; 2 Department of Microbiology, Biopathology Unit, Evgenidion Hospital, Medical School, University of Athens, Athens, GRC; 3 1st Department of Obstetrics and Gynecology, Alexandra General Hospital, National and Kapodistrian University of Athens School of Medicine, Athens, GRC; 4 Fertility Institute, National and Kapodistrian University of Athens, Athens, GRC; 5 Urology, University of Ioannina School of Medicine, Ioannina, GRC; 6 Obstetrics and Gynecology, National and Kapodistrian University of Athens, Athens, GRC

**Keywords:** assisted reproductive technology (art), in vitro fertilization (ivf), intracytoplasmic sperm injection (icsi), cell free dna, nipt

## Abstract

Background

As the offspring of assisted reproduction techniques (ARTs) have become a substantial proportion of the population, increased attention has been placed on the safety of ART. Investigators have focused on identifying a tool that combines molecular or biological tests that can predict the outcomes of in-vitro fertilization (IVF) or intracytoplasmic sperm injection and the resulting pregnancy after ART-mediated embryo implantation. This study aimed to answer the following questions: is there a difference between natural conception and IVF pregnancies regarding fetal fraction (FF) of cell-free DNA (cfDNA) in maternal age, birth weight, gender, and gestational age? Is there a difference between FF concentration regarding the parameters of IVF as possible predictive factors affecting the outcomes of IVF?

Methodology

This study included 31 women with singleton pregnancies conceived via IVF who underwent cell-free fetal DNA (cffDNA) screening for trisomy 13, 18, and 21; sex determination; and FF. The control group included 55 women who experienced natural conception. For all women, anthropometric characteristics such as age, weight, height, and body mass index (BMI) were recorded. For the IVF group, early follicular phase values of follicle-stimulating hormone, luteinizing hormone, prolactin, anti-müllerian hormone, thyroid-stimulating hormone, and estradiol were recorded.

Results

The natural conception and IVF groups were similar regarding maternal age, BMI of the mother, gender, birth weight, and gestational age. FF was not significantly different between the natural conception and IVF groups (10 (3.8) vs. 9 (2.6); p = 0.144). The results were similar after adjusting for maternal age via regression analysis. cfDNA was not associated with maternal age, birth weight, gender, or gestational age in the entire study sample or separately for the natural conception and IVF groups. No significant correlation was found between cfDNA and IVF parameters.

Conclusions

The FF is an important factor for non-invasive prenatal testing (NIPT) accuracy. Several studies have found a reduction in FF in pregnancies following ART compared with natural conception, while other studies have presented no differences in the FF. All researchers agree on the importance of NIPT; however, knowledge on how the FF is affected in ART pregnancies compared with naturally conceived pregnancies is very limited. In this study, no difference in FF for the IVF group compared with natural conception women was observed. The cffDNA concentrations in maternal serum do not appear to be affected in IVF conception. We suggest that FF is an independent factor compared with IVF parameters.

## Introduction

As children born via assisted reproduction techniques (ARTs) have become a substantial proportion of the population, increased attention has been placed on the safety of ARTs. Concerns have been raised that children conceived via ART might be exposed to greater health risks than children born of natural conception. Ovulation-induction medications, the in-vitro culture of embryos, vitrification, and the potential use of genetically and structurally abnormal sperm during intracytoplasmic sperm injection (ICSI) are independent risk factors. Recent advancements in research and practice have enabled the molecular-level examination of offspring born of ART-mediated pregnancies [1,2].

The methods for diagnosing chromosome abnormalities and screening the viability of a transfer require embryo biopsy, a procedure that affects embryo quality and requires specialized skills. The principle of non-invasive chromosome screening (NICS) has recently been demonstrated; it is based on sequencing the genomic DNA detected in the culture medium from the embryo, avoiding the need for embryo biopsy and substantially increasing safety [3,4]. Invasive prenatal testing for ART is not accepted by expecting mothers because of the low but existing miscarriage rate associated with the technique used.

Cell-free fetal DNA (cffDNA) is derived from the placenta and increases as the placenta grows [5,6]. The fetal fraction (FF) is the proportion of the maternal cell-free DNA (cfDNA) in a blood sample. A higher FF is associated with greater test sensitivity and positive predictive value (PPV) [7]. Current quantification demonstrates a median FF of approximately 11% at the time of testing [8]. While some laboratories do not report FF results, others report the test as failed if the FF is <4% [9]. The FF increases as gestational age advances, varies according to ethnicity, and is lower in women with a higher body mass index (BMI) and in pregnancies conceived via in-vitro fertilization (IVF) [10-13]. Using quantitative real-time polymerase chain reaction (PCR), high mean concentrations (6.2% of total plasma DNA) of fetal DNA were found in maternal plasma in early and late pregnancy [14].

cffDNA comprises fragments of DNA from the nucleus, a result of apoptotic or necrotic processes [15]. The plasma DNA concentration varies between 10 and 100 ng or 10^3^ and 10^4^ GE/mL [16,17]. The level of cffDNA has been determined in the bloodstream of pregnant women [18]. The technology enables the differentiation of maternal cfDNA and cffDNA when the fetus is male due to the presence of the Y chromosome. Increased cffDNA is associated with pathologies of pregnancy such as pre-eclampsia.

Investigators have focused on identifying a tool that combines molecular or biological tests that can predict the outcome of IVF or ICSI and pregnancy development after ART-mediated embryo implantation. Many tests are used in clinical practice to optimize treatment, including examining the level of follicle-stimulating hormone (FSH) and anti-Müllerian hormone (AMH), antral follicle count (AFC) by ultrasound, and genetic determination of single-nucleotide polymorphisms (SNPs) in genes such as follicle-stimulating hormone receptor (FSHR), anti-Müllerian hormone receptor (AMHR), and estrogen receptor (ESR) [19,20]. The findings of these tests are crucial, and the ultimate goal is to use routine diagnostic tests before IVF to predict factors associated with its success or failure. This endeavor can identify more cost-effective and accurate ways to promote IVF success, such as improved embryo selection to drive a healthy delivery.

If the FF is lower in IVF conceptions, the expected consequence is a higher test failure rate. The current literature on the effect of IVF conception on cffDNA testing characteristics is limited and inconclusive. Previous studies have shown no difference in the FF between IVF and naturally conceived populations [21-23]. On the other hand, Lee et al. [12] and Talbot et al. [24] demonstrated that the FF is significantly lower in IVF cases and that the test failure rate is higher compared with naturally conceived cases. In addition, the PPV of cffDNA testing is lower in singleton pregnancies conceived via IVF than those conceived spontaneously [13,24].

To investigate this discrepancy in the literature, we designed a case-control study. Our primary aim was to compare the FF and PPV of cffDNA testing in pregnancies conceived naturally and through IVF. Our secondary aim was to investigate whether there is a correlation between FF and specific IVF parameters, including the hormonal profile, ovulation-induction protocol, and embryologic profile. We recorded the maternal age, ethnicity, and BMI, as well as the gestational age, during non-invasive pregnancy testing (NIPT) to assess the homogeneity of the sample in both groups. We sought to answer the following questions: is there a difference between natural conception and ART (IVF/ICSI)-conceived pregnancy regarding the FF? Is there a difference concerning the FF and maternal age, birth weight, offspring sex, and gestational age in the total sample and separately for the natural conception and IVF groups? Is there a difference between the natural conception and IVF groups regarding maternal age divided into <35 and >35 years? Is there a difference between the FF regarding the hormonal profile, maternal age, maternal BMI, the characteristics of ovarian stimulation, the number of oocytes, the maturation rate, the fertilization rate, and the embryo quality as possible predictive factors affecting the outcome of IVF?

## Materials and methods

The study protocol was approved by the review board of the Fertility Institute. All participants provided informed consent for their medical records to be used in the study and for cffDNA testing.

The study cohort study comprised 31 women with singleton pregnancies who underwent cffDNA screening for trisomy 13, 18, and 21; sex determination; and FF. The women had undergone different reproductive modalities in a private Unit Fertility Institute. The control group included 55 women who had naturally conceived. All study participants were non-diabetic and non-smoking. For all women, anthropometric characteristics such as age, weight, height, and BMI were recorded. For the IVF group, early follicular phase values of FSH, luteinizing hormone (LH), prolactin (PRL), AMH, thyroid-stimulating hormone (TSH), and estradiol (E2) within the preceding six months were recorded.

In both groups, cffDNA testing was performed at 13 weeks of gestation using the Harmony Prenatal Test platform. For testing, 20mL samples of maternal blood were collected and sent to Ariosa Diagnostics, Inc. (San Jose, CA, USA) for analysis. The results were returned for pregnancy management and test characteristics were documented. Risk scores for aneuploidy were reported as percentages ranging from <0.01% to >99.9% or were inconclusive and no report was issued. The FF was reported as a percentage if >4%. For samples with an FF of <4%, the laboratory did not generate a risk assessment.

Controlled ovarian hyperstimulation (COH)

COH was conducted according to the gonadotropin-releasing hormone (GnRH) agonist protocol, as described previously [19]. Briefly, patients aged <35 began a long stimulation protocol. On day 21 of the previous cycle, a baseline ultrasound scan was performed and buserelin acetate intranasal spray administration was started at a dose of 100 μg five times per day. GnRH agonist administration was maintained until human chorionic gonadotropin (hCG) administration began. The extent of ovarian suppression in all patients was evaluated by ultrasound scan and serum E2 levels (<40 pg/mL) before starting exogenous gonadotrophin administration (about 15 days after administering the spray). After a follow-up, hCG was given when at least two follicles were >18 mm and serum estrogen levels were rising.

Embryos were scored and chosen for transfer based on rapid cleavage, the absence of fragmentation, and the size of the blastomeres (good quality, A; poor quality, B) [25]. Biochemical pregnancy was defined as a positive biochemical pregnancy test 18 days after oocyte retrieval. Clinical pregnancy was defined as the presence of a gestational sac on ultrasound at six gestational weeks.

Statistical analysis

Statistical analysis was performed using SPSS version 24 (IBM Corp., Armonk, NY, USA), while the Sasieni algorithm (1997) and Hardy-Weinberg equilibrium were performed using an online calculator (available at http://ihg.gsf.de). The statistical methods used for the control of the statistical hypothesis were: independent-samples t-test, two-proportion test (normal approximation), and parametric one-way analysis of variance (ANOVA). For qualitative data, the chi-squared test and Fisher’s exact test were used. The non-parametric Mann-Whitney U test and the Kruskal-Wallis test were used when needed to compare continuous variables between different groups (when the normality assumption was not satisfied). Statistical significance was set at p-values of 0.05.

## Results

Clinical characteristics

Table 1 shows the clinical characteristics of both groups. The natural conception and IVF groups were similar in terms of age, weight, BMI, sex of the offspring, birth weight, and gestational age determined by NIPT.

**Table 1:** Characteristics of the two study groups. ^+^Student’s t-test; ^‡^Pearson’s chi-square test; ^‡‡^Fisher’s exact test BMI: body mass index; IVF: in-vitro fertilization; SD: standard deviation

		Groups		P-value
		Natural conception (N = 55)	IVF (N = 31)	
		Mean (SD)	Mean (SD)	
Maternal age (years)		36.4 (3.1)	35.4 (3.8)	0.180^+^
Weight		62.3 (7.9)	59.7 (11.1)	0.242^+^
BMI		22.8 (3.0)	21.5 (3.7)	0.103^+^
BMI, N (%)				
	Underweight	1 (2.7)	3 (9.7)	0.405^‡‡^
	Normal	31 (83.8)	25 (80.6)	
	Overweight	4 (10.8)	1 (3.2)	
	Obese	1 (2.7)	2 (6.5)	
Offspring sex, N (%)				
	Male	24 (49.0)	11 (47.8)	0.927^‡^
	Female	25 (51.0)	12 (52.2)	
Birth weight		3,097.1 (334.0)	3,095.5 (421.5)	0.987^+^
Gestational age (weeks delivery)		38.5 (1)	38.0 (1.6)	0.101^+^

Comparison of FF between the natural conception and IVF group

The FF level was not significantly different between the natural conception and IVF groups. The results were similar after adjusting for maternal age via regression analysis. Regression analysis is a powerful statistical method that allows the examination of the relationship between two or more variables of interest. It was performed to gain more power for the statistical analysis as patients’ age is a crucial factor in the NIPT procedure (Table 2).

**Table 2:** Comparison of the FF between the two study groups: FF (%) in natural conception (NC) and IVF. ^++^Comparison of FF between groups after adjusting for maternal age. IVF: in-vitro fertilization; FF: fetal fraction; SD: standard deviation; SE: standard error

	Groups		P-value		
	Natural conception	IVF			
	Mean (SD)	Mean (SD)		(SE)^++^	Ρ^++^
FF (%)	10 (3.8)	9 (2.6)	0.173+	-1.18 (0.80)	0.144

The women were further categorized according to an FF cut-off of 6% (Table 3). There was no difference between the natural conception and IVF groups based on this classification.

**Table 3:** Comparison of the FF between the two study groups: FF (%) >6 and <6 in natural conception versus IVF. FF: fetal fraction; IVF: in-vitro fertilization

		Groups				
		Natural conception		IVF		
		N	%	N	%	Fisher’s exact test (P)
FF (%)	≤6	9	16.7	3	9.7	0.522
	>6	45	83.3	28	90.3	

Finally, the FF of the two groups was similar when maternal age was divided into <35 and >35 years (Table 4).

**Table 4:** Comparison of the FF between the two study groups: FF (%) in age >35 versus <35. IVF: in-vitro fertilization; FF: fetal fraction; SD: standard deviation

				FF		
				Mean	SD	Student’s t-test (P)
Groups	Normal conception	Age	≤35	10.4	4.6	0.823
			>35	10.1	3.4	
	IVF	Age	≤35	9.1	3.3	0.819
			>35	8.9	1.8	

Clinical characteristics of the total sample and separated into the natural conception and IVF groups

There were no significant correlations between the FF and the maternal age, birth weight, sex of the offspring, or gestational age when considering the total sample or the natural conception and IVF groups separately (Tables 5, 6).

**Table 5:** Correlation between the FF and maternal age, birth weight, offspring sex, and gestational age in the total sample and separately for the natural conception and IVF groups: FF (%) in the total sample versus natural conception and IVF. ^+^Pearson’s correlation coefficient; ^++^Student’s t-test IVF: in-vitro fertilization; FF: fetal fraction; SD: standard deviation

		FF (%)					
		Total sample		Natural conception		IVF	
		Mean (SD)	P-value	Mean (SD)	P-value	Mean (SD)	P-value
Maternal age (years)							
	≤35	9.8 (4.0)	0.934^++^	10.4 (4.6)	0.823^++^	9.1 (3.3)	0.819^++^
	>35	9.7 (3.0)		10.1 (3.4)		8.9 (1.8)	
Maternal age (years), r^+^		0.08	0.466	0.05	0.730	0.08	0.671
Sex of the offspring							
	Male	9.6 (3.2)	0.409^++^	9.6 (3.3)	0.211^++^	9.5 (3.2)	0.551^++^
	Female	10.2 (3.7)		10.9 (4.1)		8.8 (2.3)	
Birth weight, r^+^		-0.11	0.380	-0.14	0.382	-0.07	0.758
Gestational age (weeks), r^+^		0.13	0.314	0.10	0.549	0.15	0.521

**Table 6:** Correlation between the FF and maternal age: FF (%) in the total sample of age >35 and <35 in the natural conception and IVF groups. IVF: in-vitro fertilization; FF: fetal fraction; SD: standard deviation

			FF		
			Mean	SD	P-value
Age (years)	≤35	Natural conception	10.4	4.6	0.355
		IVF	9.1	3.3	
	>35	Natural conception	10.1	3.4	0.189
		IVF	8.9	1.8	

The FF and hormonal profile of the IVF group

In the IVF group, there were no significant correlations between the FF and the levels of hormones (βhCG, FSH, LH, PRL, TSH, and AMH). In addition, there were no significant correlations between the FF and the IVF parameters on the day of ovulation, E2 on the day of hCG administration, the number of embryos, and the morphological quality of embryos (Table 7). There were also no significant correlations between the βhCG change and the level of other hormones, the day of stimulation, E2 on the day of hCG administration, and the number of embryos (Table 8).

**Table 7:** Correlation between the FF and the level of hormones and IVF parameters. ^+^Spearman’s correlation coefficient; ^++^Pearson’s correlation coefficient AMH: anti-Müllerian hormone; E2: estradiol; FF: fetal fraction; FSH: follicle-stimulating hormone; hCG: human chorionic gonadotropin; LH: luteinizing hormone; TSH: thyroid-stimulating hormone

	FF (%)	
	r	P-values
βhCG change	-0.12^+^	0.512
A-βhCG	-0.28^+^	0.120
B-βhCG	-0.19^+^	0.312
FSH	-0.08^++^	0.659
LH	-0.25^++^	0.182
Prolactin	0.07^+^	0.728
TSH	-0.01^++^	0.937
Days of stimulation	0.10^+^	0.620
E2 on the day of hCG	-0.13^++^	0.532
No embryos	-0.11^++^	0.541
Embryo quality	0.21^+^	0.269
ΑΜΗ	0.01	0.975

**Table 8:** Correlation between βhCG and IVF parameters. ^+^Spearman’s correlation coefficient. AMH: anti-Müllerian hormone; E2: estradiol; FF: fetal fraction; FSH: follicle-stimulating hormone; hCG: human chorionic gonadotropin; LH: luteinizing hormone; TSH: thyroid-stimulating hormone

	βhCG change	
	r^+^	P-values
FSH	0.10	0.580
LH	-0.30	0.096
Prolactin	0.28	0.132
TSH	0.10	0.576
Days of stimulation	0.00	0.988
E2 on the day of hCG	0.21	0.295
No embryos	0.10	0.582
Embryo quality	-0.32	0.080
ΑΜΗ	-0.11	0.574

Comparison of FSH, LH, PRL, and AMH with FF

The correlation between FSH, LH, PRL, and AMH levels and FF was non-significant. Concerning the comparison regarding the IVF parameters, we obtained the following results: for FSH, a level of >8 IU/L was associated with patients aged >35. A mean PRL level of >11.0 pg/mL was associated with a higher mean βhCG level. A higher E2 level on the day of hCG administration and higher oocyte collection were noted when the AMH level was >3 ng/mL. These results did not affect the FF.

One case of Down syndrome was recorded in the IVF group. The mother had the following characteristics: 35 years old, weight 65 kg and height 1.68 m, with a 2.5-year period of infertility due to a tubal factor; FF of 4% and a PPV of 76.5%; first βhCG of 795 IU/L and second βhCG of 1,736 IU/L; FSH of 6.6 IU/L, LH of 8.6 IU/L, TSH of 2.68 mIU/L, anti-TPO of <9 IU/mL, anti-TG of <10 IU/mL, and AMH of 6.69 ng/mL; short protocol implemented with rFSH, eight days of ovulation, amount of gonadotropins 1,575 IU, E2 on the day of hCG 3,299 pg/mL, 11 oocytes harvested, 10 oocytes fertilized, two embryos transferred, on day five blastocysts.

## Discussion

Although FF testing is considered to be a primary screening test, only a few studies have assessed its performance, especially among patients undergoing ART. Among women achieving pregnancy via ART maternal anxiety may lead to hesitancy in undergoing this test. Moreover, in the contemporary literature, there are contradictory results regarding the PPV of the FF in patients undergoing ART. Some studies have reported no significant contribution of the method of conception [21-23], while others have observed a decreased FF in pregnancies conceived via IVF [13,24].

Age, ethnicity, BMI, and gestational age are critical components of FF testing. Hence, in this study, we matched the participants in the natural conception and IVF groups for age, ethnicity (Caucasian), BMI, weeks of pregnancy when NIPT was performed, sex of the offspring, birth weight, and gestational age. According to our results, there were no significant differences between the natural conception and IVF groups in these clinical parameters (Table 1). The FF of the two study groups was not significantly different (Table 2). The results were similar after adjusting for maternal age via regression analysis. Furthermore, when categorized according to a cut-off point of 6% (Table 2), the FF was not significantly different between the natural conception and IVF groups. Moreover, the FF was not significantly different between the natural conception and IVF groups when the mothers were categorized according to age (>35 and <35 years). The FF was not associated with maternal age, birth weight, sex of the offspring, or gestational age in the total sample or separately for the natural conception and IVF groups (Table 3). While we have analyzed a relatively homogenous population, the restrictive criteria have limited the sample size.

Lee et al. and Talbot et al. reported that the FF is significantly lower in pregnancies conceived via IVF than those conceived spontaneously [12,24]. They suggested that a lower FF increased the test failure rate and decreased the PPV in IVF-mediated versus spontaneous conceptions. These findings have implications for pre-test counseling provided to women conceiving via IVF. When comparing the demographic data of Lee et al. and our current data, there are differences in age, BMI, and weight [12]. For example, Lee et al. reported different mean ages for the spontaneous conception and IVF groups (33.8 and 36.6 years, respectively) and differences in ethnicity (61.2% and 83.7% Caucasian, respectively) [12]. The heterogeneous sample in their study could explain the low FF in the IVF group. In the study by Talbot et al., the control group included high-risk pregnancies based on the combined first-trimester screening, so these women had a high risk for trisomy 21 [24]. The authors found a reduction in the FF in pregnancies following fresh compared with frozen embryo transfer. They hypothesized that this reduction in the FF is due to the compromised placental formation following ovarian stimulation in fresh embryo transfers. This observation is in contrast to the study by Lee et al. who did not observe any difference between fresh and frozen embryos regarding FF [12].

We have investigated the proteomic and metabolomic profile of children born following ART compared with naturally conceived controls to identify epigenetic abnormalities [1,2]. We found that ART likely causes some epigenetic changes in the offspring, which might be the molecular basis of complex traits and diseases. In this context, we examined the correlation between the FF and several parameters, including hormones, maternal age, maternal BMI, type of gonadotrophins, characteristics of ovarian stimulation, embryologic profile, the number of oocytes, the maturation rate, the fertilization rate, and the quality of embryos, to determine possible predictive factors affecting the outcome of IVF/ICSI. There were no significant correlations between the FF and hormones. Women with FSH levels of >8 IU/L were older and women aged >35 more often presented FSH levels of >8 IU/L. Women with PRL of >11 pg/mL also presented higher levels of βhCG. Women with AMH of >3 ng/mL presented a significantly higher level of E2 on the day of hCG administration and more oocytes.

These results did not affect the FF. Indeed, the FF does not appear to have any association with the IVF profile and is an independent factor concerning IVF parameters.

In the literature, studies have used cffDNA as an additional serum marker (e.g., Down syndrome screening) without adjustment in IVF pregnancies. IVF does not affect levels of cffDNA, which appears to be independent of traditional screening markers (e.g., hCG). Pan et al. [21] showed that the cffDNA level in the maternal serum does not appear to be affected by IVF conception and, therefore, may not need adjustment for pregnancies achieved via IVF compared with natural conceptions.

Lambert-Messerlian et al. observed that ART-mediated pregnancies and natural conceptions contribute similar levels of circulating cffDNA into the maternal circulation [22]. Costa et al. reported that examining cffDNA performed better than maternal serum screening in both spontaneous and ART-mediating pregnancies, thus decreasing the number of invasive procedures [23]. However, these studies clearly present results that do not show an increase in circulating cffDNA in pregnancies achieved using ART, either in absolute levels or based on the FF. Our findings are consistent with the absence of an increase in the amount of cffDNA in maternal plasma from pregnancies conceived via IVF compared with natural conception.

Even though Lee et al. found a reduction in FF in patients undergoing IVF, they reported that 97.6% of cffDNA tests in IVF pregnancies provided a result regarding trisomy 21, but the failure rate is higher, the FF is lower, and the PPV for trisomy 18 and 13 and sex chromosome abnormality is decreased in IVF pregnancies compared with those conceived spontaneously [12]. They recommend that these limitations should be taken into account during pre-test counseling in pregnant women who conceive via IVF.

Talbot et al. showed a significant reduction in the FF in patients submitted to ART compared with patients who conceived naturally; the difference appeared to be more pronounced after fresh compared with frozen embryo transfer. Lee et al. [12] did not make this observation in frozen embryos, where the FF was similar in the fresh and frozen groups [27].

The FF is an important factor for NIPT test accuracy. Several studies have found a reduction in FF for pregnancies following ART compared with natural conception, while others have presented no differences in the FF. All researchers agree on the importance of NIPT. The most important issue is that even with a reduction in FF (97.6%), cffDNA tests for IVF pregnancies give accurate results regarding trisomy 21 [13]. However, knowledge on how the FF is affected in ART pregnancies compared with naturally conceived pregnancies is very limited.

Of course, ART-mediated pregnancies are different compared to natural conception for several reasons. The cause of infertility of the parents, the embryo culture media, and COH have been shown to influence the imprinting status of some genes [28,29]. Indeed, epigenetic changes during the preimplantation period could be a potential mechanism for altered growth, development, and metabolism of ART-conceived children. More specifically, concerns have been raised about the overall health of children born via IVF/ICSI as this method has a greater risk for the introduction of genetic errors by bypassing all intrinsic barriers for the fertilization of abnormal gametes, thus eliminating sperm natural selection. The parameters for a successful NIPT result in natural conceptions are BMI, ethnicity, gestational age, maternal weight, and maternal height. On the other hand, in ART-mediated pregnancies, there are additional variables that play a crucial role in NIPT. Thus, we need to consider the culture media, the ovulation-induction protocol, and the stage of embryo transfer on day three or five. Considering these factors, it appears very difficult to design a study with homogeneous material that would provide the true picture of the evaluation of NIPT in women who have undergone ART.

## Conclusions

We found no difference in the FF for the natural conception and IVF groups. The FF in maternal serum does not appear to be affected in IVF conception. There were no correlations between the FF and IVF parameters. Thus, we suggest that the FF is an independent factor compared with IVF parameters.
